# Sound transmission in a bamboo forest and its implications for information transfer in giant panda (*Ailuropoda melanoleuca*) bleats

**DOI:** 10.1038/s41598-018-31155-5

**Published:** 2018-09-20

**Authors:** Benjamin D. Charlton, Megan A. Owen, Jennifer L. Keating, Meghan S. Martin-Wintle, Hemin Zhang, Ronald R. Swaisgood

**Affiliations:** 1 San Diego Zoo’s Institute for Conservation Research, California, CA 92027-7000 USA; 2 China Conservation and Research Center for the Giant Panda, Dujianyan, Sichuan China

## Abstract

Although mammal vocalisations signal attributes about the caller that are important in a range of contexts, relatively few studies have investigated the transmission of specific types of information encoded in mammal calls. In this study we broadcast and re-recorded giant panda bleats in a bamboo plantation, to assess the stability of individuality and sex differences in these calls over distance, and determine how the acoustic structure of giant panda bleats degrades in this species’ typical environment. Our results indicate that vocal recognition of the caller’s identity and sex is not likely to be possible when the distance between the vocaliser and receiver exceeds 20 m and 10 m, respectively. Further analysis revealed that the F0 contour of bleats was subject to high structural degradation as it propagated through the bamboo canopy, making the measurement of mean F0 and F0 modulation characteristics highly unreliable at distances exceeding 10 m. The most stable acoustic features of bleats in the bamboo forest environment (lowest % variation) were the upper formants and overall formant spacing. The analysis of amplitude attenuation revealed that the fifth and sixth formant are more prone to decay than the other frequency components of bleats, however, the fifth formant still remained the most prominent and persistent frequency component over distance. Paired with previous studies, these results show that giant panda bleats have the potential to signal the caller’s identity at distances of up to 20 m and reliably transmit sex differences up to 10 m from the caller, and suggest that information encoded by F0 modulation in bleats could only be functionally relevant during close-range interactions in this species’ natural environment.

## Introduction

The acoustic structure of mammal vocal signals encodes information about the caller that has functional relevance in a range of behavioural contexts (for a review see^1^). However, the distances over which specific acoustic information can potentially be transmitted in a given species’ natural environment are often unknown. This is somewhat surprising because effective transmission of acoustic signals is highly dependent on the characteristics of the environment. For example, sound waves are subject to ~6 dB loss of amplitude per doubling of distance as they propagate through the environment (termed ‘spherical spreading’) and additional ‘excess’ attenuation caused by reverberation, ground attenuation, and atmospheric disturbances such as wind and rain^2–4^. In addition, because sound frequencies do not attenuate in a consistent fashion across the frequency spectrum^3,5–9^, specific frequency components that are important for categorising callers could be more or less attenuated than others^2,10,11^. Hence, being able to detect a vocal signal above the environmental background noise does not equate to being able to extract functionally relevant information from calls. Determining how the spectral composition and information content of vocalisations degrades as it propagates through the environment is therefore a pre-requisite for gaining a complete understanding of the function of different vocal signals, because it allows researchers to determine the type of information that animals could potentially extract from calls at different distances

Giant pandas (*Ailuropoda melanoleuca*) are Ursidae mammals that inhabit the bamboo forests of south central China. Because these animals live a solitary existence, effective communication is likely to be crucial for locating other conspecifics and coordinating mating activities during the annual reproductive period^12–14^. The coordination of courtship prior to direct encounters appears to be primarily mediated through olfactory signals, allowing receivers to recognise kin^15^, and determine the identity^16^, sex^17^, reproductive state^18^, and competitive status of signallers^19,20^. However, giant pandas also have a diverse vocal repertoire that is likely to be important for promoting contact between individuals and mediating close-range interactions during the breeding season^21,22^. In particular, male giant pandas produce bleat vocalisations at high rates when they encounter oestrous females^14^ and following investigation of oestrous female odours^17^ indicating that these calls are important for coordinating this specie’s mating activities.

Recent studies have used a source-filter theory^23^ approach to show that giant panda bleats contain a wide range of reliable information about the caller. According to the source-filter theory mammal vocalisations consist of a fundamental frequency (F0), which reflects the rate of vocal fold vibration in the larynx, and formants that correspond to the resonance frequencies of air in the supra-laryngeal vocal tract^1^. In giant panda bleats mean F0, the level of amplitude modulation, and the upper formants encode information on the caller’s identity^24^ and higher rates of F0 modulation are indicative of males with higher androgen levels^25^. In addition, formant frequency spacing is a reliable cue to male body size in this species^26^ because larger males have longer vocal tracts, which produce lower, more closely spaced formants. Since male giant pandas are roughly 15–20% larger than females^26^, males also have significantly lower formant spacing than females, making this acoustic feature a reliable cue to the caller’s sex^26^. Playback studies have also shown that giant pandas attend to this potentially important information during the breeding season^27–29^. More recent work has revealed that male giant pandas produce longer duration bleats with higher mean F0 during vocal interactions with peak oestrous females^30^, indicating that F0 is also a cue to the caller’s motivational state in this species.

While the information content of giant panda bleats is well documented, the precise function of these calls is less clear. Outside of the breeding season giant pandas occupy distinct but overlapping seasonal ranges^31,32^ and, although rare, interactions among individuals are often highly aggressive when they occur^13^. During the breeding season male and female giant panda home ranges expand^32,33^, locomotor and signalling activity increases^34^, and females’ deposition of oestrous urine attracts multiple males that compete for access to fertile females^13,18,35^. In addition, male giant pandas appear to avoid areas scent marked by more competitive males^19^. Because bleats are individually distinctive vocalisations^24^ they could be used to identify and avoid conspecifics that are known to have been victorious in previous agonistic encounters, thereby helping to reduce the chances of significant, and potentially fatal injury. During the breeding season identity cues in bleats may even attain crucial importance for maintaining contact between courting individuals in dense bamboo thickets where there is limited opportunity for visual contact. Females could also use acoustic cues to the caller’s identity to become progressively familiarised to the vocalisations of high-quality males that are able to outcompete other rivals and maintain close contact with them during the lead up to mating, and subsequently go on to prefer these individuals in mate choice contexts^29^. In support of this contention, free-ranging male giant pandas are known to compete for access to females during the breeding season, and will often associate with females for up to one month before copulation and subsequent separation^13,35,36^. Female giant pandas seeking a mate may also be able to extract other useful information from bleats over distance. For example, information about the caller’s sex could allow unreceptive (non-oestrous) females to better avoid potentially aggressive roaming males during the breeding season, and females could use the rate of F0 modulation in bleats to assess male androgen levels and sexual motivation in mate choice contexts. The wide-range of information encoded in bleats cannot, however, be functionally relevant unless it is reliably transmitted in the giant panda’s bamboo forest environment.

The aims of the current study were twofold: 1) to establish how far individual and sex differences in giant panda bleats could realistically be broadcast in a bamboo forest environment; and 2) to examine how bleat acoustic structure degrades over distance. In line with the findings of previous sound propagation studies in forest environments^3,4^, we predicted that the frequency components of giant panda bleats below 1 kHz would be the least stable over distance. We also expected the amplitude of higher frequencies to attenuate more rapidly than lower frequencies^37–39^. Our findings will afford insights into the distances over which giant pandas could potentially signal important information, and could help conservation biologists to estimate population levels using non-invasive bioacoustic techniques.

## Methods

### Experimental site and weather conditions

To examine how the acoustic structure of giant panda bleats degrades with distance, we played back 100 bleats (10 from each of 10 adult giant pandas) and re-recorded them at distances of 10, 20, 30, and 40 metres (m) away from the playback speaker. The playback and re-recording of giant panda bleats took place in a mixed bamboo plantation at San Diego wild animal park, California, United States. The plantation had a density of 40–50 bamboo culms per sqM and consisted of *Phyllostachys vivax*, *P*. *aureasulcata* and *P. bambusoides* species. This bamboo density is well within the range of bamboo density in panda habitat, which varies from ~10–140 culms per sqM, depending on species, geographic location, slope and other variables^40–43^. The density of the bamboo thicket was fairly heterogenous with leaves and branches remaining thick down to around 50 cm. We played back and recorded bleats between 0400–0600 on the 28th August 2015 and 1st April 2016. The recordings captured on the 1^st^ April 2016 contained considerably more ambient noise than those captured on the 28^th^ August 2015. Accordingly, for the current analysis we chose to analyse and present the results obtained using bleats re-recorded on the 28th August 2015.

For the two-hour recording session on the 28th August the mean temperature was 14.6 degrees Celsius (range = 14–16 C), the humidity varied between 75–79% and there was very little wind (<4 kph, measured using a Siemens anemometer). The average temperature in the Foping Nature Reserve, Qinling mountains, Sichuan Province, China, which lies within the northernmost part of the giant pandas natural range, during the 2018 breeding season (Feb-June) was 14.6 degrees Celsius, and the relative humidity varied between 14–100% (taken from https://www.timeanddate.com). The sound propagation experiments in the current study were therefore conducted at a temperature and humidity that is realistic for this species’ natural environment.

### Test sounds

The original recordings of adult giant panda bleats used as test sounds in the transmission experiments were obtained during the 2008 and 2009 breeding seasons from two individuals at Chengdu Research Base of Giant Panda Breeding, Chengdu, China (Bing Dien, Cheng Cheng), six individuals at the China Research and Conservation Centre for the Giant Panda, Bi Feng Xia nature reserve, Sichuan, China (Fei Fei, Lu Lu, Wu Gang, Xi Xi, Ying Ying and Yuan Yuan), and two individuals resident at Zoo Atlanta (Lun Lun), and San Diego Zoo (Gao Gao) in the United States. This gave us a set of recordings from 10 adult giant pandas, five male and five female, with ages ranging from 6–21 years (mean = 11.5). All the animals were individually recognizable and housed separately.

Giant panda bleats were recorded with a Sennheisser ME67 directional microphone connected to a TASCAM HDP2 or a Marantz PMD660 digital recorder at distances of 1–5 m from the caller, and with no physical obstacles to impede sound transmission. The recordings were transferred to an Apple Macbook Pro laptop computer so that they could be normalized to 100% peak amplitude and stored as WAV files (44.1 kHz sampling rate and 16 bits amplitude resolution). We then selected 10 recordings with low levels of background noise from each of the 10 giant pandas to use in the transmission experiments. Because the original recording distance (<5 m) was below the minimum re-recording distance of 10 m, any potential affects of signal degradation between the animal and the microphone were minimized.

### Playback and re-recording of test sounds

A Chiayo Focus 505 loudspeaker (Taipei, Taiwan) with a frequency response of 50–15000 Hz (±3 dB) was used to playback the giant panda bleats used as test sounds in the transmission experiment. The loudspeaker was calibrated to broadcast bleats at mean sound pressure levels of 75 dB 1 meter from the source (measured using a Radio Shack Sound Level Meter, set for C-weighted fast response), sounding equivalent to naturally bleating giant pandas^27–29^. Bleats were rerecorded using a RODE NTG-2 directional microphone (frequency range: 20–20000 Hz, sensitivity −36dB ± 2 dB) fitted with a RODE windshield attached to a Zoom H4N digital recorder at a sampling rate of 44.1 kHz, and an amplitude resolution of 16 bits. The use of directional microphones helps to limit the effect of ground reflections on the re-recordings^3^.

Giant pandas typically bleat whilst they are walking with all four limbs in contact with the ground, and data from Davis on giant panda body proportions^44^ indicates that the distance between the ground and the midline of the skull, and therefore the mouth and ears of a vocalising animal, is approximately 80 cm. Thus, to mimic natural calling height in this species we used tripods to place the microphone and loudspeaker at a vertical height of 80 cm above the ground. For each re-recording distance the speaker and microphone remained oriented towards one another. The speaker was in a fixed location while the microphone was moved, and we used a Bushnell Yardage Pro laser rangefinder to mark out the re-recording distances prior to the experiment. Finally, we re-recorded the test sounds 1 m from the speaker to control for potential acoustic changes due to the broadcasting equipment. These were then used as reference (‘model’) sounds with which to compare the transmitted (‘observation’) sounds in the subsequent analysis.

### Acoustic analyses

Praat 6.0.31 DSP package^45^ was used to conduct the acoustic analyses on reference and re-recorded (transmitted) bleats To carry out the analysis in an objective fashion we used custom-built programs to automatically extract and measure a range of acoustic measures before logging these variables in an output file. These outputs were then checked against relevant spectrograms and power spectrum to ensure Praat was accurately tracking and measuring all acoustic features.

**Figure 1:**
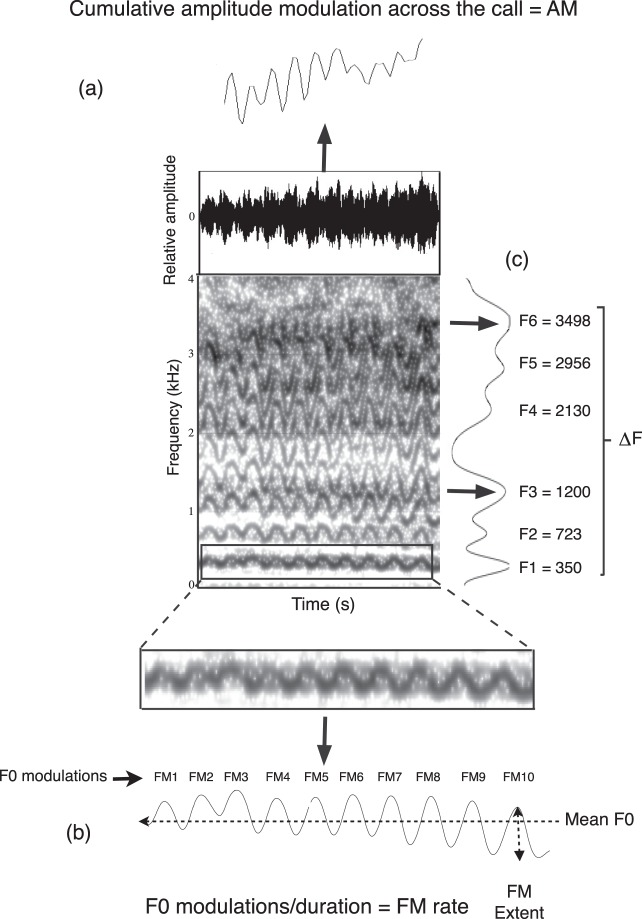
Waveform and spectrogram of a giant panda bleat to illustrate the acoustic measures on. (**a**) The intensity contour (AM); (**b**) the F0 contour (mean F0, FM extent, FM rate); and (**c**) the frequency spectrum (F1, F2, F3, F4, F5, F6 and ∆F). Spectrogram settings: FFT method; window length 0.05 s; time step = 0.004 s; frequency step = 10 Hz; Gaussian window shape; dynamic range = 50 dB. Bleats are characterised by F0 and amplitude modulation. Reproduced from Charlton *et al*. J. Acoust. Soc. Am. 126(5), 2721 (2009), with the permission of the Acoustical Society of America.

The mean F0 values for each bleat were extracted using a forward cross-correlation (‘To F0 (cc)’ command) algorithm in Praat. The time step in the analysis was 0.01 seconds, and the minimum and maximum values for F0 were set at 200 Hz and 850 Hz, respectively. A five point average smoothing filter removed any rapid variations in the F0 contour before the mean F0 value across the entire bleat was determined using the ‘get mean’ command in Praat. In addition, to quantify the characteristic F0 modulation of giant panda bleats, we measured the number of complete cycles of fundamental frequency modulation per second (FM rate) and the average peak-to-peak variation of each fundamental frequency modulation (FM extent) (Fig. 1). The intensity contour of each bleat was also extracted (‘To intensity’ command) to measure the average modulation per second in dB (AM) (Fig. 1).

Linear Predictive Coding (LPC; ‘To Formants (Burg)’ command in Praat) was used to measure the formant frequencies of giant panda bleats. The frequency values of the first six formants were extracted using following analysis parameters: time step: 0.01 seconds; window analysis: 0.2 seconds; maximum formant value: 3800–4000 Hz; maximum number of formants: 5–6; pre-emphasis: 50 Hz. To accurately measure the lower 3 formants we ran an additional analysis with the following parameters: time step: 0.01 seconds; window analysis: 0.2 seconds; maximum formant value: 2000 Hz; maximum number of formants: 3; pre-emphasis: 50 Hz, and formants 1–3 from the first analysis were discarded. The formant spacing (∆F) during each bleat was then calculated using the linear regression method of Reby and McComb^46^. Descriptive statistics for all of the acoustic measures are provided in Table 1.

**Table 1:** Descriptive statistics for the acoustic measures of reference bleats re-recorded at 1 m (N = 100).

Acoustic measures	*M*	s.d.	Minimum	Maximum
F0 (Hz)	405.9.7	134.4	263.0	772.5
FM extent (Hz)	119.6	44.5	43.0	242.0
FM rate (cps)	8.4	1.9	5.0	14.0
AM (dB)	143.6	63.8	57.7	343.9
F1 (Hz)	435.6	40.5	363.0	545.0
F2 (Hz)	503.8	112.5	334.0	842.0
F3 (Hz)	1334.4	87.2	1138.0	1548.0
F4 (Hz)	2211.1	169.8	1890.0	2586.0
F5 (Hz)	3168.0	116.8	2820.0	3379.0
F6 (Hz)	3564.7	131.3	3155.0	3852.0
∆F (Hz)	573.7	32.0	502.0	636.0

Hz = Hertz, cps = cycles per second, dB = decibels.

### Discriminant analyses

To evaluate individual and sex differences in the acoustic structure of giant panda bleats Discriminant Function Analyses (DFA) were used to classify calls at the different re-recording distances (1 m, 10 m, 20 m, 30 m, and 40 m), with either subject identity or sex entered as the group identifier, and the 11 acoustic measures entered as discriminant variables. Both the re-classification and more conservative leave-one-out cross-validation procedure were applied, and to ensure the robustness of the classification we pooled the results from 1000 bootstrap samples and used bias-corrected and accelerated confidence intervals (using the ‘Bootstrap…’ option in SPSS). For each acoustic measure missing values were replaced by the mean value obtained for the corresponding re-recording distance so that equal group sizes could be compared. There were no missing acoustic values for bleats rerecorded at 1 m, 10 m, and 20 m. A total of 13 out of 50 bleats rerecorded at 30 m, and 40 out of 50 bleats at 40 m had at least one missing acoustic value. To assess the stability of individual and sex differences in bleats over distance, we used the ‘hold-out-sample’ method^47^ in which the acoustic measures of relatively un-degraded bleats re-recorded at 1 m were used to classify observation bleats re-recorded at 10 m, 20 m, 30 m, and 40 m to the different individuals and the caller’s sex. For each of the 10 individuals in the analysis we used 5 reference bleats to train the models to classify the five remaining bleats from each individual re-recorded at each distance. Thus, different bleats were used for training and classification sensu^48^. IBM SPSS statistics version 20 was used to run the DFAs and the Chi square statistic allowed us to determine whether correct classification attained statistical significance,

### Analysis of bleat degradation over distance

**Figure 2:**
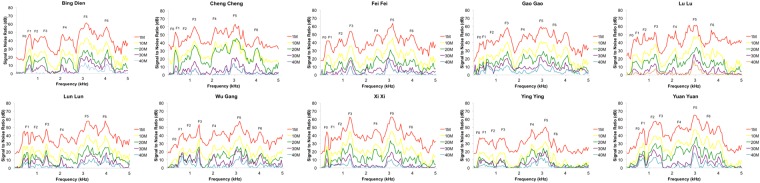
Signal-to-Noise-Ratio plots. The plots illustrate how the frequency components of giant panda bleats from 10 individuals attenuate over distance.

To examine the stability of different acoustic measures we calculated the absolute percentage (%) difference between the acoustic measures of reference bleats re-recorded at 1 m and the acoustic measures from the same bleats at the different re-recording distances. To assess amplitude attenuation we computed a long-term average spectrum (LTAS) for one bleat from each of the 10 giant pandas at each distance (1 m, 10 m, 20 m, 30 m, and 40 m). We were then able to visually assess the amplitude attenuation across the frequency spectrum averaged across the call. Bleats were down-sampled to 10 kHz and a frequency analysis window of 50 Hz was used to generate the LTAS, so that 100 quantitative variables were produced, each one giving the amplitude (dB) of a 50-Hz frequency slice up to 5000 Hz. To calculate the Signal to Noise Ratio (SNR) for each bleat, LTAS of a 1 s segment of background noise immediately following an observation bleat were subtracted from the bleat LTAS, thereby controlling as far as possible for any background noise present in the call (see Fig. 2). To examine amplitude degradation we extracted the peak SNR for F0 and F1-F6 at the re-recording distances, and then regressed these values for each of the 10 giant panda bleats with re-recording distance. This allowed us to calculate regression slope values and quantify the amplitude degradation for each of these individual frequency components over distance. We then compared the amplitude curves with the expected amplitude loss due to spherical spreading, of 6 dB per doubling of distance^4^, to determine whether excess attenuation was occurring.

## Results

### Discriminant analyses

#### Individual classification of giant panda bleats over distance

The DFA correctly classified 94% of bleats re-recorded at 1 m to caller identity (81% cross-validated). This level of classification was statistically significant compared to that expected by chance (Table 2). Hold-out-sample DFA’s trained with bleats re-recorded at 1 m were able to classify 60% of bleats rerecorded at 10 m, 40% of those rerecorded at 20 m, 14% for those rerecorded at 30 m, and 16% of those rerecorded at 40 m (Table 2). Bleats re-recorded at distances of 10 m and 20 m were classified to the 10 different giant pandas significantly above chance levels (Table 2). In contrast, the classification rates for bleats re-recorded at 30 m and 40 m did not attain statistical significance (Table 2).

**Table 2:** Percent (%) observed correct classification to caller identity and sex against expected levels.

Identity					Sex			
Distance (m)	Expected	Observed	*X* ^2^	*P*	Expected	Observed	*X* ^2^	*P*
1 (test)	10%	94%	705.6	**<0**.**01**	50%	89%	30.4	**<0**.**01**
10	10%	60%	250.0	**<0**.**01**	50%	76%	13.5	**<0**.**01**
20	10%	40%	90.0	**<0**.**01**	50%	58%	1.3	0.25
30	10%	14%	1.6	0.99	50%	44%	0.0	1.00
40	10%	16%	3.6	0.94	50%	50%	0.0	1.00

Bleats re-recorded at 1 m were used to train DFAs to classify bleats re-recorded at 10 m, 20 m, 30 m, and 40 m to each of the 10 giant pandas (‘hold-out-sample’ method). The Chi square statistic (*X*^2^) was used to determine the statistical significance of correct classification.

#### Classification of the caller’s sex over distance

89% of bleats re-recorded at 1 m were correctly attributed to the caller’s sex, falling to 79% when a more conservative leave-one-out cross validation was applied. Compared to that expected by chance this level of classification was statistically significant (Table 2). Hold-out-sample DFA’s trained with bleats re-recorded at 1 m classified 76% of bleats to the caller’s sex at 10 m, 58% of bleats to the caller’s sex at 20 m, 44% of bleats to the caller’s sex at 30 m, and 50% of bleats to the caller’s sex at 40 m (Table 2). The level of classification was statistically significant at 10 m, but not at 20 m, 30 m, and 40 m.

### Analysis of bleat degradation over distance

#### Stability of acoustic measures

Mean % variation for all the acoustic measures increased as the distance between the speaker and microphone increased (Table 3), demonstrating that vocal characteristics became less reliable over distance. The upper formants F4, F5 and F6 had the lowest % variation values (Table 3), and hence, were the most stable acoustic measures. Mean F0 also had relatively low % variation when the distance between speaker and microphone was 20 m or less (mean variation of 2.1%), but became very unreliable as the distance increased to 30 m and beyond. FM rate, FM extent, AM and F2 also had relatively high % variation values (Table 3), indicating that these acoustic features were relatively unstable over distance.

**Table 3:** Absolute % difference between reference and observation bleats at the different re-recording distances.

Acoustic measures	Mean % variation in acoustic values at each distance				
	10 m	20 m	30 m	40 m	Mean
Mean F0	1.3	2.1	28.2	46.5	19.5
FM extent	1.2	15.4	12.4	29.8	14.7
FM rate	2.2	19.4	45.3	53.3	30.0
AM	8.9	17.4	20.2	28.1	18.6
F1	2.0	5.4	7.4	5.3	5.0
F2	11.4	25.7	28.8	30.5	24.1
F3	1.6	1.9	2.4	3.3	2.3
F4	0.3	0.8	0.2	0.8	0.5
F5	1.3	1.7	0.4	0.8	1.1
F6	0.8	1.0	0.6	0.3	0.7
∆F	0.5	1.7	2.7	3.5	2.1

The acoustic measures of bleats re-recorded over distances of 10–40 m were compared with the acoustic measures of reference bleats re-recorded at 1 m.

#### Amplitude attenuation

**Figure 3:**
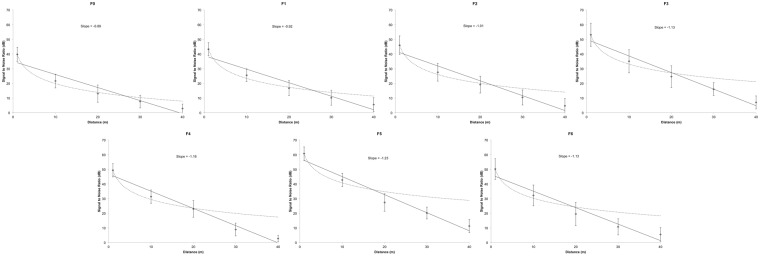
Mean Signal-to-Noise-Ratio values and regression lines for individual frequency components at the different distances. The regression slope values quantify how the frequency amplitudes of re-recorded bleats drop over distance. Higher regression slope values indicate greater amplitude attenuation over distance. The dotted lines represent the predicted attenuation via spherical spreading. The error bars show the standard deviation of the mean SNR values for each of the 10 giant pandas in the analysis. F5 and F3 were the highest amplitude formants in the reference bleats re-recorded at 1 m, and remained so over distance.

The SNR plots indicate that individual frequency components (F0 and F1-F6) degrade in a relatively uniform manner as they propagate through the *bamboo* canopy (Fig. 2). The SNR regression plots show that excess attenuation (i.e. above the expected 6 dB drop off per doubling of distance) occurs for all frequency components of bleats at re-recording distances of ~30 m and above, and at distances of ~20 m and above for F5 and F6, making these frequency components the most prone to decay (Fig. 3).

**Figure 4:**
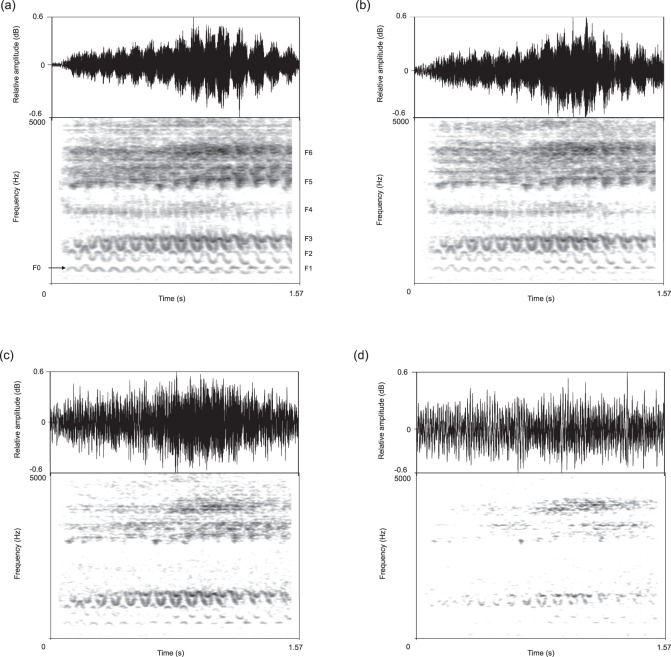
Waveforms and spectrograms to show how the acoustic structure of a bleat degrades over distance. (**a**) Re-recording at 10 m; (**b**) re-recording at 20 m; (**c**) re-recording at 30 m; (**d**) re-recording at 40 m. F0 and formants (F1–F6) are labelled in re-recording at 10 m (**a**).

Although F5 is subject to relatively high amplitude attenuation in the bamboo forest environment it still remains the most prominent and persistent frequency component of bleats, with a SNR of 60.7 dB at 1 m, dropping 11.3 dB at 40 m from the speaker (Fig. 3). In contrast, F4 and F0 are only just above the background noise at 40 m (Fig. 3) and, therefore, unlikely to be perceptible to receivers at this distance. The spectrograms in Fig. 4 also show how the acoustic structure of a single bleat from one of the 10 giant pandas degrades over distance. While F3, F5 and F6 are still visible in the spectrogram at 40 m, the F0 contour becomes almost completely immersed in background noise at this distance, making the measurement of F0 characteristics extremely unreliable.

## Discussion

In the current study we have shown that the acoustic structure of giant panda bleats remains individually distinctive over distances of up to 20 m in a bamboo forest environment. Other mammal studies using similar techniques to ours revealed that individual vocal distinctiveness is reliably propagated in species-typical environments over distances ranging from 16 m to 1000 m^11,49–51^. The ability of swift foxes (*Vulpes velox*)^51^ and African elephants (*Loxodonta africana*)^11^ to acoustically signal identity over distances of 400 m to 1000 m, respectively, is probably due to the open terrain or savannah habitats these species typically inhabit^11,51^, in which acoustic signals are often less distorted by reverberations than those produced in forest or closed environments^39^. The literature for forest-dwelling mammals reveals that Bornean orangutan (*Pongo pygmaeus*) long calls remain individually distinctive over distances of up to 300 m^38^, and koala (*Phascolarctos cinereus*) bellows reliably transmit individually distinctive vocal characteristics over distances of up 50 m^48^. Both of these arboreal species, however, deliver and receive calls from the forest canopy several metres above the ground^38,48^, which generally reduces reverberation and the attenuation of acoustic signals when compared to vocal signal transmission from approximately ground level^52^. Giant panda bleats therefore appear to remain individually distinctive over an expected range when this species’ typical bamboo forest habitat and calling position are taken into consideration.

In addition, while our results indicate that giant pandas are unlikely to *recognize* known individuals at distances greater than 20 m, bleats could still be used to detect the presence of a conspecific over longer range, so that individuals could then choose to approach and gain more information about the caller. Furthermore, different environmental conditions will affect the distances over which identity cues in bleats could be reliably transmitted. For example, sound propagation distances are increased when there is less ambient noise, and when humidity and temperature are lower^2^. Since the current study was conducted at a temperature and humidity that is typical for the giant panda’s natural environment, and with almost no wind noise, we suggest that the results presented here provide an accurate reflection of giant panda bleat transmission characteristics in species-typical propagation conditions.

Previous giant panda studies have shown that the main contributors to acoustic individuality in bleats are mean F0 and AM^24^. Further studies revealed that giant pandas could discriminate between the bleats of different male callers when F0 was held constant across individuals but not when AM was removed^29^, indicating that AM is fundamentally important for vocal discrimination. Hence, degradation of amplitude modulation characteristics is likely to be a key factor constraining vocal recognition to distances of 20 m or below in a bamboo forest environment. Consistent with this interpretation, we found that mean % variation in AM exceeded 20% at distances of 30 m and above, which would most likely compromise any vocal recognition process based on this acoustic feature of bleats. Mean F0 also became highly unreliable at distances of 30 m and 40 m, indicating that this acoustic feature is also very unlikely to be used to discriminate between callers at distances ≥30 m.

Our findings indicate that vocal recognition and the ability to vocally signal individual identity may only attain importance when individuals are within 20 m of one another. At these distances, the ability for male giant pandas to distinguish between the bleats of unfamiliar and familiar same-sexed rivals could facilitate the avoidance of direct, and potentially aggressive, encounters^53^, and females would have the opportunity to familiarise themselves with the vocalisations of certain males that can outcompete other rivals and maintain close proximity to them in the lead up to oestrus^29^. Recent work suggests that female giant pandas do assess potential mates over a longer timeframe than their 1–3 day oestrous period^54^, and familiarity with a given male’s vocalisations may form part of this assessment. It is also likely that giant pandas use chemical signals to determine the identity of conspecifics^16^. We suggest that information acquired from chemical signals might be linked to individually distinctive vocal characteristics when close contact has been established. This type of cross-modal integration of information occurs in other mammals^55–57^ and could allow giant pandas to develop a sensory construct of different individuals, and thus, display true ‘recognition’.

It is also worth noting that our results are reliant on accurate extraction and measurement of vocal parameters using Praat, and the statistical tests used to interpret acoustic variation, which may differ from the capability of giant pandas to extract acoustic information from bleats. Indeed, it is very likely that giant pandas incorporate additional acoustic features, such as the formants, into the vocal recognition process. In addition, giant pandas could learn to *recognize* the bleats of certain individuals at distances of 30 m and above by focussing on other individually distinctively acoustic features (such as F5 and ∆F) that remain stable over distance (as found in zebra finches^58^). Habituation–discrimination playback studies have shown that giant pandas can discriminate between the bleats of different callers at close range^29^. A similar approach is now required to determine whether they can discriminate between different callers over distances of 20 m or more.

Our results also suggest that giant pandas would be unable to use bleats to categorise individuals as male or female over distances greater than 10 m. FM extent and ∆F are reliable cues to the caller’s sex in giant panda bleats^26^. In the present study we found that mean FM extent became very unreliable at distances greater or equal to 20 m, making it extremely unlikely that giant pandas could use this acoustic feature to assess the caller’s sex over these distances. In addition, although ∆F remained highly stable out to 40 m, it is unlikely to be sufficient for determining the caller’s sex from a distance because mean ∆F for male and female giant pandas only differs by 11 Hz^26^, equating to a 1.7% difference between the sexes. Since we found that variability in ∆F rises to 1.7% and above at distance greater than 10 m, we suggest that any sex differences in this acoustic feature would almost certainly be obscured when the distance between signaller and receiver exceeds 10 m. It would appear then, that the rapid decay of FM characteristics in a bamboo forest environment, along with the relatively small sex difference in ∆F, constitutes the main reason why re-classifying bleats to the caller’s sex dropped to chance levels at 10 m and beyond.

As predicted, the most unreliable frequency components of bleats over distance were all under 1 kHz. Structural degradation of the F0 contour would make it especially difficult for conspecific receivers to reliably assess FM rate and FM extent over distances greater than 10 m. Because FM rate and FM extent are cues to male giant panda androgen levels and motivational state, respectively^25,30^, these findings indicate that information on male hormonal quality and arousal state in giant panda bleats would not be propagated reliably over distance in a bamboo forest environment. The poor stability of F0 characteristics could be attributed to frequency-dependent reverberation^2^. Reverberation occurs when sound waves are reflected or scattered and then later re-join the main beam of sound propagation^4^ and it is considerably greater in forests than in open areas due to reflections from tree trunks and foliage^2^. In addition, previous studies that broadcast artificial signals in forest environments have shown that reverberation over distance is especially pronounced for frequencies below 1 kHz^39,52^. Accordingly, since the mean F0 of giant panda bleats is around 400 Hz, this observation may explain why F0 characteristics are less stable than other frequency components of bleats above 1 kHz. The giant pandas’ natural propagation height of ~80 cm^44^ could also make frequencies below 1 kHz relatively unreliable over distance due to ground reflections^3^.

The analysis of bleat degradation indicates that F3, F5 and F6 were subject to more severe amplitude attenuation than other frequency components as they propagate though the bamboo forest environment. These findings are consistent with our prediction that the higher frequencies of bleats would attenuate at greater rates than lower frequencies^2,3^. The relatively high source intensity of F5, however, appears to counteract the environmental degradation of this feature, which remains 11.3 dB above background noise at 40 m. F5 is the most highly individualised feature of bleats after F0 and AM^24^. Consequently, the high source amplitude of this formant may represent an adaptation to facilitate the propagation of individual vocal distinctiveness in bleats. F5 is around 3.2 kHz in giant panda bleats^24^, which is approximately the same frequency as a prominent spectral peak found in the voices of trained opera singers, termed the ‘singer’s formant’^59,60^. The ‘singer’s formant’ is produced when the epi-laryngeal tube, the airspace between the vocal folds and the aryepiglottic folds, acts as a separate resonator, due to its aperture being much narrower than the cross-sectional area of the pharynx^61^. Imaging studies are now required to determine whether the giant panda has evolved a specialized epi-laryngeal tube that is compatible with the production of a high amplitude formant at around 3 kHz. Interestingly, a recent behavioural test of the giant panda’s auditory capabilities also revealed increased hearing sensitivity around 500 Hz and 2000 Hz^62^, which corresponds approximately with the lowest amplitude frequency components of bleats at 40 m, F0 and F4. Hence, this species’ hearing sensitivity may also reflect selection pressures to perceive information encoded by F0 and F4 in the contexts of identity cueing.

These findings also shed new light on giant panda reproductive strategies by demonstrating the likely range over which acoustic communication in support of breeding can take place. It has been previously speculated that olfactory communication is important for mate location and signalling the onset of female oestrous, whereas acoustic signals are better suited for late-oestrus motivational coordination between pairs^12^. Our findings suggest that most acoustic communication does indeed take place over very short distances (10–20 m) once mates have been located. Further, observations of mating aggregations have revealed that dominant male giant pandas remain in close proximity to oestrus females while subordinate males are typically 10–50 m away. These distances may therefore limit female acoustic access to subordinate males, thwarting mate choice mechanisms^63,64^ and putting subordinate males at a reproductive disadvantage. An additional interesting possibility is that female giant pandas select habitat that may enhance the transmission of acoustic information over short to medium ranges (<20 m). Giant pandas typically aggregate for mating in more open areas which would facilitate the transmission of acoustic signals^13,65^. Females also have a tendency to climb trees just before their fertile period, thereby elevating themselves above the bamboo understory, and, potentially increasing the distance at which they can discriminate important vocal characteristics affording information on sex, identity, and reproductive condition.

Finally, our findings may also have utility for determining population sizes in this reclusive species^13^. Identifying individuals using visual cues from camera trap images is potentially difficult because giant pandas have relatively invariant physical features^66^; however, since bleats are individually distinctive and produced at high rates when animals interact during the breeding season^14,24^, bio-acoustic monitoring of giant panda population sizes is a genuine possibility. The giant panda’s breeding and communication system lends itself to this approach. Compared with other *Ursidae*, giant pandas have relatively small home ranges^67^, and they concentrate their activities into fairly predictable locations during the mating season. Males make particular use of established communal scent mark stations typically located on ridges, and individuals visit the same sites repeatedly^68,69^. In addition, several (4–6) male giant pandas will congregate around and compete for access to an oestrous female, often in traditional breeding grounds that can be used repeatedly^35^. Guided by the results of the current study, we suggest that sound recorders are positioned 30 m apart at known giant panda breeding areas and communal scent stations, so they can reliably capture acoustic variation within bleats that can retrospectively be used to classify these calls to different individuals. Although sex differences in bleats do not propagate as well as identity cues, it may also be possible in some cases to determine the sex of different individuals from the acoustic structure of bleats. In addition, bleats should be classified to individuals using the most reliable individually distinctive features. The results of this study indicate that F5 might be a good candidate for classifying bleats to different callers because it is individually distinctive and propagates well in the giant panda’s natural environment (i.e. high stability and persistence over distance). Given the high rates of giant panda vocal activity during the breeding season it may be possible to obtain enough good quality recordings for bioacoustic techniques to be used to help estimate population sizes, aiding management and conservation efforts in this endangered species.

## Data Availability

The datasets generated during and/or analysed during the current study are available from the corresponding author on reasonable request.
